# Acute Ischemic and Hemorrhagic Stroke in COVID-19: Mounting Evidence

**DOI:** 10.7759/cureus.10157

**Published:** 2020-08-31

**Authors:** Kartikeya Rajdev, Shubham Lahan, Kate Klein, Craig A Piquette, Meilinh Thi

**Affiliations:** 1 Pulmonary and Critical Care Medicine, University of Nebraska Medical Center, Omaha, USA; 2 Internal Medicine, University College of Medical Sciences, Delhi, IND; 3 Internal Medicine, University of Nebraska Medical Center, Omaha, USA

**Keywords:** covid-19, stroke, complication, mortality, co-morbidity, intracerebral hemorrhage

## Abstract

The novel coronavirus disease of 2019 (COVID-19) is caused by the binding of severe acute respiratory syndrome coronavirus-2 (SARS-CoV-2) to angiotensin-converting enzyme 2 (ACE2) receptors present on various locations such as the pulmonary alveolar epithelium and vascular endothelium. In COVID-19 patients, the interaction of SARS-CoV-2 with these receptors in the cerebral blood vessels has been attributed to stroke. Although the incidence of acute ischemic stroke is relatively low, ranging from 1% to 6%, the mortality associated with it is substantially high, reaching as high as 38%. This case series describes three distinct yet similar scenarios of COVID-19 positive patients with several underlying comorbidities, wherein two of the patients presented to our hospital with sudden onset right-sided weakness, later diagnosed with ischemic stroke, and one patient who developed an acute intracerebral hemorrhage during his hospital stay. The patients were diagnosed with acute stroke as a complication of COVID-19 infection. We also provide an insight into the possible mechanisms responsible for the life-threatening complication. Physicians should have a low threshold for suspecting stroke in COVID-19 patients, and close observation should be kept on such patients particularly those with clinical evidence of traditional risk factors.

## Introduction

The novel coronavirus disease of 2019 (COVID-19) caused by the severe acute respiratory syndrome coronavirus-2 (SARS-CoV-2) has infected more than 20 million people causing more than 700,000 deaths worldwide as on August 14, 2020 [1]. Although the virus predominantly affects the lower respiratory tract, the extrapulmonary manifestations are not uncommon. The commonly involved systems are cardiovascular, gastrointestinal, hematological, hepatocellular, renal, and dermatological [2,3]. Another equally concerning feature of COVID-19 is the involvement of the central nervous system, which may vary from headaches, dizziness, hypogeusia, or encephalitis owing to direct viral invasion of neural tissue, to cerebrovascular disease or stroke [2,4]. The incidence of both acute ischemic stroke and hemorrhagic in COVID-19 patients is reported to range from 1% to 6% [5-10]. The acute brain infarction secondary to stroke is of particular importance because it not only substantially affects the prognosis of the patient but can also have long-term residual neurological deficits. In our case series, we present three relatively uncommon cases of COVID-19 presenting with stroke.

## Case presentation

Case 1

**Figure 1:**
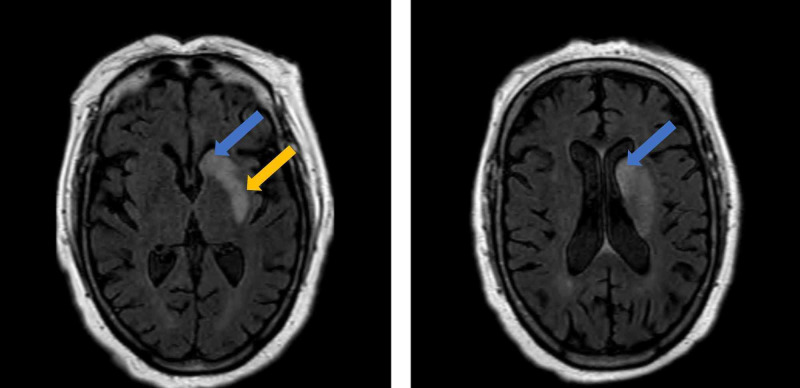
MRI showing an acute/sub-acute infarct involving the left caudate nucleus (blue arrow) and putamen (yellow arrow).

A 76-year-old woman with a past medical history of type-II diabetes mellitus, chronic obstructive pulmonary disease (COPD) on nocturnal 3 L/min home oxygen, obstructive sleep apnea on BiPAP (bilevel positive airway pressure) at home, hypertension, and dyslipidemia was diagnosed with COVID-19 infection eight days prior to her admission. She presented to the emergency department (ED) with a sudden onset of right-sided weakness and aphasia. The patient received thrombolysis therapy with tissue plasminogen activator (tPA) for ischemic stroke after a CT scan of the head was negative for hemorrhage. The patient was then transferred to our hospital for a higher level of care and evaluation for possible thrombectomy. In our ED, the patient had blood pressure (BP) and heart rate (HR) of 148/58 mmHg and 79 beats/min, respectively, with 95% SpO_2_ on 4 L of oxygen through a nasal cannula. She was awake, alert, and following commands. A critical neurologic examination revealed severe global aphasia, dense right hemiparesis, and a left gaze preference, with an NIH stroke score (NIHSS) of 13. Her initial laboratory reports did not show any derangements. The patient underwent a CT angiogram of head and neck and CT perfusion scan of the brain, both of which did not reveal any major vessel occlusion or a perfusion defect. She was then admitted to the COVID ICU and was subsequently started on aspirin and statin, and intravenous medications to maintain systolic BP < 180/105. She was also started on remdesivir for COVID-19 related illness. MRI of the brain was performed the next day that showed an acute/sub-acute infarct involving the left caudate nucleus and putamen (Figure 1).

On day 3 of admission, she was observed to have worsening somnolence (NIHSS of 19), for which another head CT scan was performed that did not reveal any new findings. Over the course of the next three days, the patient continued to deteriorate with worsening lethargy and a progressive decline in her mental status. The patient was intubated for airway protection. Despite appropriate treatment and mechanical ventilatory support, the patient’s clinical condition continued to deteriorate. The patient’s family was updated regarding her clinical prognosis, and after an adjudicated discussion, the patient’s family decided that they no longer wish to subject her to further aggressive interventions, which were also in concordance with the patient’s wishes. The following day she was compassionately extubated receiving comfort measures only. Our patient expired six days into her hospital admission.

Case 2

A 60-year-old Caucasian male with a past medical history of coronary artery disease with a two-vessel coronary artery bypass graft nine years ago, heart failure, hypertension, dyslipidemia, type 2 diabetes mellitus, methamphetamine abuse, and tobacco abuse was brought to the ED after being found down at a friend’s house. His last-known well time was the night prior to the presentation. The patient upon presentation was lethargic with severe aphasia with an HR of 123 beats/min, BP of 112/88 mmHg, temperature of 37.2°C, and oxygen saturation of 76% on room air. Upon examination, he was noted to have right-sided hemiparesis, right-sided facial droop, expressive aphasia, severe dysarthria, and left gaze preference. While in the ED, the patient became more obtunded, requiring intubation for airway protection.

**Figure 2:**
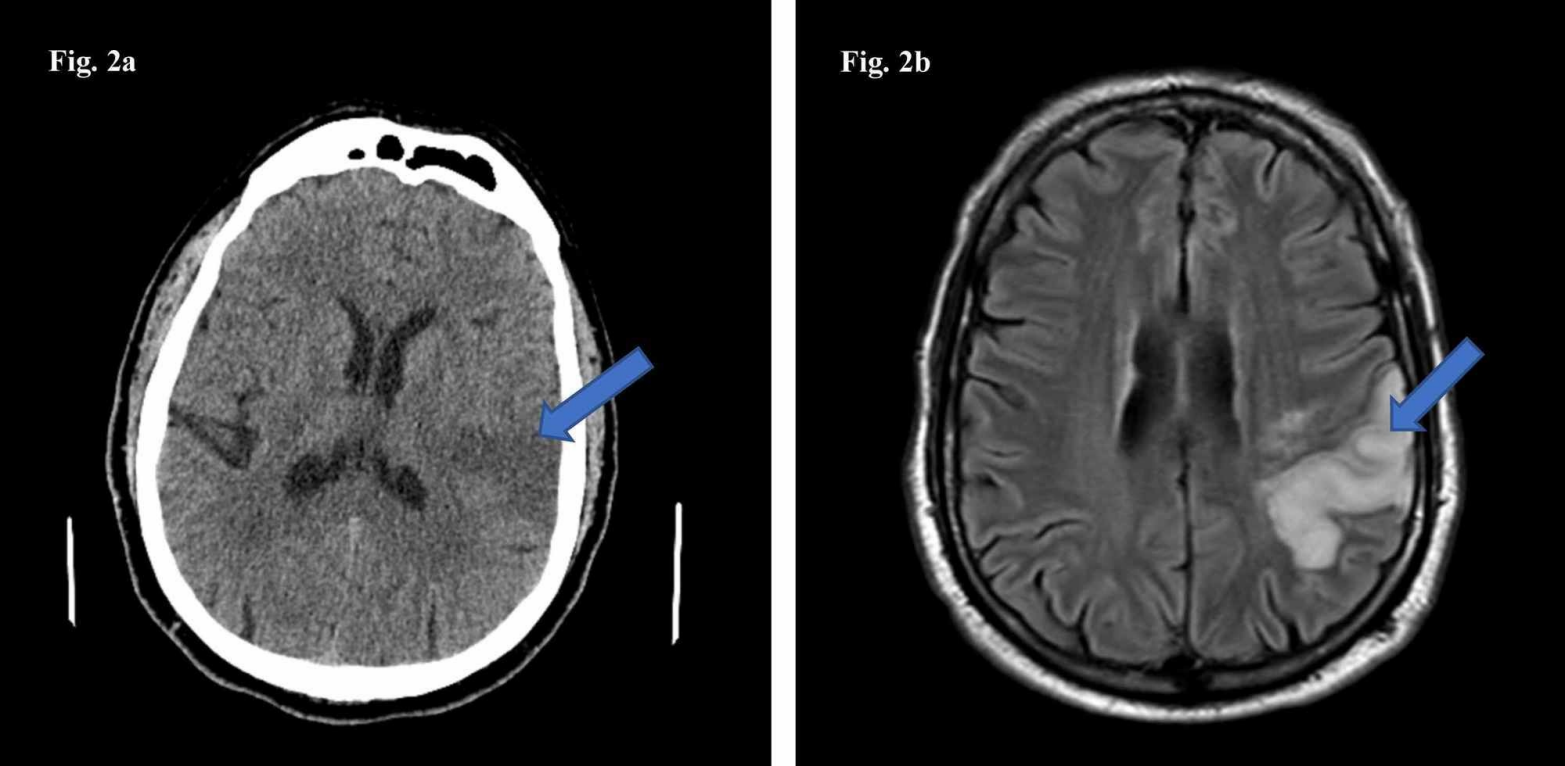
CT scan of the head (2a) and MRI of the brain (2b) showing an evolving moderate-sized subacute left MCA branch infarct (arrows).

**Figure 3:**
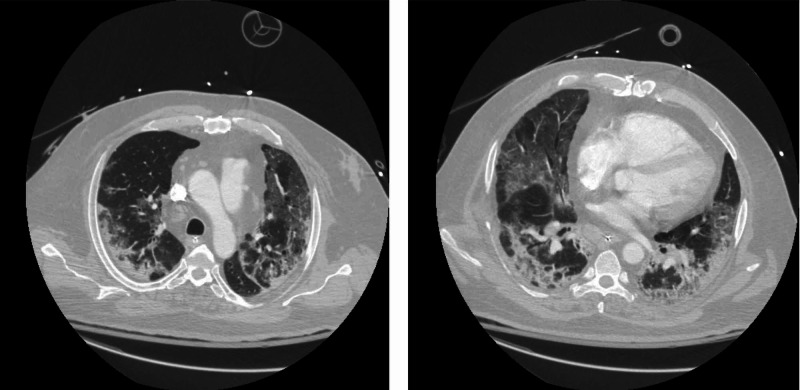
Chest CT illustrating diffuse bilateral ground-glass consolidation of lungs.

The patient had a white blood cell count of 12,500/µL, lactic acidosis of 2.9 mmol/L, and an elevated procalcitonin of 0.82 ng/mL. His chest X-ray showed bilateral patchy opacities. A CT angiogram of the head and neck revealed a moderate-sized subacute left middle cerebral artery (MCA) branch infarct, which was primarily perisylvian with a corresponding perfusion defect (Figure 2a). There was no intracranial hemorrhage, mass effect, or any major vessel occlusion. The patient was not a candidate for thrombolysis and was started on aspirin and atorvastatin. His chest CT did not show pulmonary emboli but diffuse bilateral ground-glass consolidation (Figure 3). A nasopharyngeal swab for COVID-19 PCR (polymerase chain reaction) was obtained, which came back negative. Due to the concern for COVID-19 pneumonia, a tracheal aspirate was sent for COVID-19 PCR that returned positive. The patient was initiated on remdesivir and dexamethasone for COVID-19 pneumonia. The pneumonia panel from tracheal aspirate showed methicillin-sensitive *Staphylococcus aureus*, *Streptococcus agalactiae*, and *Streptococcus pneumoniae* for which he was initiated on appropriate antibiotics. His echocardiogram showed normal systolic function with an ejection fraction (EF) of 55%. MRI of the brain showed an evolving moderate size subacute left MCA branch infarct (Figure 2b). EKG did not reveal any cardiac arrhythmia. The patient improved from a respiratory standpoint and was extubated to high-flow nasal cannula on day 8 of his hospital stay. The patient was awake, alert, and oriented; however, he continued to have right-sided hemiparesis and expressive aphasia.

Case 3

**Figure 4:**
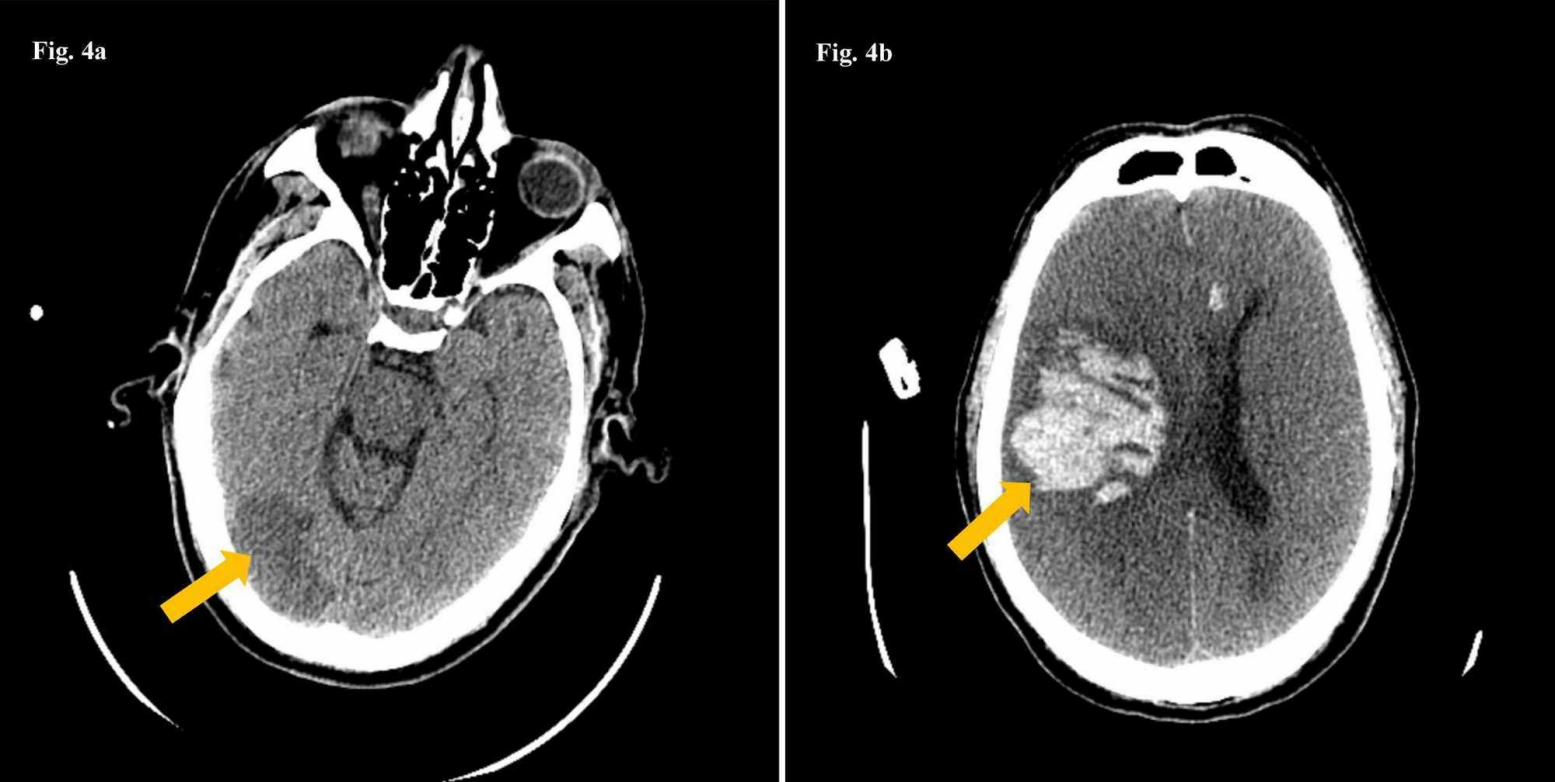
CT scan of the head with a low-density area in the right occipital lobe (4a) consistent with a subacute ischemic stroke (arrow); and right intraparenchymal hematoma (arrow) with midline shift with compression of the lateral ventricle and scattered subarachnoid hemorrhage (4b).

A 62-year-old Hispanic male with a past medical history of hypertension and tobacco abuse presented to the ED with a two-day history of progressively worsening dyspnea and cough. His vitals upon presentation were as follows: HR of 110/min, BP of 105/74 mmHg, temperature of 37.2°C, and SpO_2_ of 87% on room air. He had a leukocyte count of 19,000/µL, lactic acid level of 1.4 mmol/L, C-reactive protein of 33.2 mg/L, creatinine of 2.29 mg/dL, and an elevated procalcitonin of 6.48 ng/mL. CT scan of the chest showed bilateral nodular ground-glass opacities with a crazy-paving appearance. A nasopharyngeal swab for COVID-19 PCR was positive. The patient was also initiated on antibiotics for concomitant bacterial pneumonia with a positive *Streptococcus pneumoniae* urine antigen. His oxygen requirements gradually worsened requiring intubation on day 3 of hospitalization and vasopressor support for shock. A pneumonia panel from tracheal aspirate detected *Streptococcus pneumoniae*. He also developed worsening renal function requiring renal replacement therapy. The patient was noted to develop paraplegia on minimal sedation on day 10 of hospitalization prompting a CT scan of the head, which showed a 4.3-cm low-density area in the right occipital lobe consistent with a subacute ischemic stroke (Figure 4a). He was started on aspirin and atorvastatin. A transthoracic echocardiogram showed an EF of 65-70% with no wall motion abnormalities. His mental status continued to deteriorate until he became unresponsive with bilaterally dilated pupils. A repeat CT scan of his head showed a new large 6.0-cm right intraparenchymal hematoma with associated midline shift and transtentorial herniation with evidence of global hypoxic-ischemic injury and herniation-related infarction as well as scattered subarachnoid hemorrhage (Figure 4b). The patient was receiving subcutaneous heparin for deep vein thrombosis (DVT) prophylaxis but no therapeutic anticoagulation was given. In the best interest of the patient and in accordance with the patient’s wishes, the family decided to transition his goals of care to comfort care only. The patient expired after a prolonged hospital stay of 27 days.

## Discussion

Largely due to its multi-system involvement and a wide spectrum of clinical presentations, COVID-19 continues to pose challenges affecting clinical prognosis, particularly among the elderly and those with underlying comorbidities, such as our patients. The clinical profiles of our patients are consistent with the findings of a recent meta-analysis by Tan et al. who concluded that the mean duration of an acute ischemic stroke from the onset of COVID-19 symptoms was 10 ± 8 days, with a mean age of 63.4 ± 13 years [8].

The precise mechanism responsible for causing acute ischemic stroke in COVID-19 positive patients is currently unclear. However, proposed speculations suggest that the process may be multifactorial. The SARS-CoV-2 gains entry into the human body by attaching itself to the membrane-bound angiotensin-converting enzyme 2 (ACE2) receptors located on, but not limited to, pulmonary alveolar epithelial cells and vascular endothelium [2,11]. The viral interaction with ACE2 receptors in blood vessels causes endothelial damage and induces a hyperimmune response: “cytokine storm” releasing inflammatory markers such as interleukin (IL)-1, IL-6, and tumor necrosis factor-alpha (TNF-a) that might predispose to stroke by causing vasculitis [2,3,12]. Moreover, COVID-19 is also associated with hypercoagulability and hyperviscosity, which can immensely increase the risk of stroke [13,14]. Lastly, the pre-existence of traditional risk factors such as hypertension, dyslipidemia, diabetes, and atherosclerotic vascular disease (such as in our case) in the setting of extensive COVID-19 infection may have a synergistic contribution in increasing patient predisposition to stroke by causing plaque disruption. The ACE2 receptors also play a role in the pathogenesis of hemorrhagic stroke, wherein the downregulation of receptors coupled with elevated angiotensin II and endothelial dysfunction in cerebral arteries increases BP, and thus the risk of hemorrhage [4]. Pre-existing hypertension is a potential risk factor for hemorrhagic stroke.

Stroke (both ischemic and hemorrhagic) occurring in the setting of COVID-19 is reported to have a worse patient prognosis, with a substantially higher risk of in-hospital mortality [9]. One study found that the risk of mortality among COVID-19 patients with ischemic stroke was 32% [7]. Tan et al. also reported a similar finding in their meta-analysis, with a cumulative mortality risk of 38% [8]. Strategies that seem plausible in treating COVID-19 patients with stroke include the use of tPA, but the role of other anticoagulants such as low-molecular-weight heparin or unfractionated heparin is debatable and controversial. Since SARS-CoV-2 binds to and depletes ACE2 receptors, administration of exogenous ACE2 in the form of human recombinant soluble ACE2 (hrs ACE2) might reduce the risk of acute stroke by replenishing ACE2 in cerebral blood vessels [13,15].

Interestingly, elevated serum levels of D-dimer, fibrinogen, and anti-phospholipid antibodies are implicated in COVID-19 patients with ischemic stroke [8]. Periodic assaying of these biomarkers might help in identifying patients with increased stroke predisposition. Workflow proposed by Qureshi et al. seems promising as it lays down a step-by-step guide for the management of acute ischemic stroke for the physicians [16]. Another study involving 10 patients with ischemic stroke reported that early intravenous thrombolysis and recanalization through mechanical thrombectomy had resulted in poor outcomes in their patient cohorts [17]. Moreover, stroke teams need to be extra cautious so as to minimize the risk of self-exposure with the proper use of protective equipment.

## Conclusions

Both ischemic and hemorrhagic stroke are associated with COVID-19 infection and carry a high risk of mortality. The ACE2 receptors, circulating cytokines, and hypercoagulability are integral in the pathogenesis of stroke in COVID-19 patients. Further studies need to be conducted to understand the exact mechanism in COVID-19 patients with stroke. However, treating physicians should have a low threshold for suspecting stroke in COVID-19 patients, and close observation should be kept on such patients particularly those with clinical evidence of traditional risk factors.
